# A tractometry principal component analysis of white matter tract network structure and relationships with cognitive function in relapsing-remitting multiple sclerosis

**DOI:** 10.1016/j.nicl.2022.102995

**Published:** 2022-03-24

**Authors:** Danka Jandric, Geoff J.M. Parker, Hamied Haroon, Valentina Tomassini, Nils Muhlert, Ilona Lipp

**Affiliations:** a Division of Neuroscience & Experimental Psychology, School of Biological Sciences, Faculty of Biology, Medicine and Health, University of Manchester, Manchester Academic Health Science Centre, Manchester, UK; b Centre for Medical Image Computing, Department of Medical Physics & Biomedical Engineering and Department of Neuroinflammation, Queen Square Institute of Neurology, University College London, London, UK; c Bioxydyn Limited, Manchester, UK; d Cardiff University Brain Research Imaging Centre, Cardiff University, Cardiff, UK; e Institute for Advanced Biomedical Technologies (ITAB), Department of Neurosciences, Imaging and Clinical Sciences, University G. d’Annunzio of Chieti-Pescara, Chieti, Italy; f Multiple Sclerosis Centre, Department of Neurology, SS. Annunziata University Hospital, Chieti, Italy; g Department of Neurophysics, Max Planck Institute for Human Cognitive & Brain Sciences, Leipzig, Germany

**Keywords:** Multiple sclerosis, Cognitive impairment, MRI, Tractometry, Brain connectivity

## Abstract

Understanding the brain changes underlying cognitive dysfunction is a key priority in multiple sclerosis (MS) to improve monitoring and treatment of this debilitating symptom. Functional connectivity network changes are associated with cognitive dysfunction, but it is less well understood how changes in normal appearing white matter relate to cognitive symptoms. If white matter tracts have network structure it would be expected that tracts within a network share susceptibility to MS pathology. In the present study, we used a tractometry approach to explore patterns of variance in white matter metrics across white matter (WM) tracts, and assessed how such patterns relate to neuropsychological test performance across cognitive domains. A sample of 102 relapsing-remitting MS patients and 27 healthy controls underwent MRI and neuropsychological testing. Tractography was performed on diffusion MRI data to extract 40 WM tracts and microstructural measures were extracted from each tract. Principal component analysis (PCA) was used to decompose metrics from all tracts to assess the presence of any co-variance structure among the tracts. Similarly, PCA was applied to cognitive test scores to identify the main cognitive domains. Finally, we assessed the ability of tract co-variance patterns to predict test performance across cognitive domains. We found that a single co-variance pattern which captured microstructure across all tracts explained the most variance (65% variance explained) and that there was little evidence for separate, smaller network patterns of pathology. Variance in this pattern was explained by effects related to lesions, but one main co-variance pattern persisted after this effect was regressed out. This main WM tract co-variance pattern contributed to explaining a modest degree of variance in one of our four cognitive domains in MS. These findings highlight the need to investigate the relationship between the normal appearing white matter and cognitive impairment further and on a more granular level, to improve the understanding of the network structure of the brain in MS.

## Highlights

• Elucidating the neural correlates of cognitive impairment in MS is a research priority for improving symptom management.
• Tractometry was used to identify patterns of white matter pathology in MS.
• Results showed a single pattern of co-variance across white matter tracts.
• Variance in normal appearing white matter significantly contributes to cognitive domains in MS.
• Findings question shared susceptibility among tracts to pathology and highlight need for further research.

## 1 Introduction

The ‘clinico-radiological paradox’ (Barkhof, 1999, Barkhof, 2002) highlights the poor correlation between demyelinating lesions and clinical symptoms in multiple sclerosis (MS). Cognitive symptoms are common and associated with poor outcomes, but the pathology is poorly understood (Sumowski et al., 2018), making cognitive dysfunction a challenge in the management of MS. A research priority is therefore to identify MRI correlates of cognitive impairment to understand pathological mechanisms better.

Cognition in MS is often evaluated as a global impairment, yet cognitive dysfunction involves deficits in separate domains, including processing speed and memory (Charcot, 1877, Benedict et al., 2006, Sepulcre et al., 2006, Migliore et al., 2016, Matias-Guiu et al., 2017, De Meo et al., 2021). Understanding if and how different cognitive domains are susceptible to different underlying brain abnormalities can inform our understanding of the mechanisms of cognitive impairment in MS.

Cognitive symptoms in MS have been associated with functional network connectivity abnormalities (reviewed in Chard et al., 2021, Jandric et al., 2021a, Jandric et al., 2021b), but the mechanisms causing these functional connectivity changes are not known. White matter (WM) damage can influence functional connectivity (Schoonheim et al., 2015, Patel et al., 2018, Tewarie et al., 2018), possibly through alteration of anatomical connections between functionally connected regions (Catani and ffytche, 2005, Dineen et al., 2009). Such WM damage could be due to lesions or to pathological processes in tissue outside of lesions. There is evidence of both secondary axonal loss from inflammatory activity in lesions, such as Wallerian degeneration, and lesion-independent degeneration of axons following demyelination resulting from more diffuse inflammation in non-lesioned tissue (Trapp et al., 1998, Bitsch et al., 2000, Trapp and Stys, 2009).

Diffusion MRI studies (dMRI) have established associations between cognitive impairment and damage to non-lesioned, or normal-appearing white matter (NAWM) in MS. These studies have largely used whole-brain analyses of the WM, such as tract-based spatial statistics (TBSS, Smith et al., 2006), to show that non-lesional damage in specific WM areas, such as the corpus callosum and cingulum, correlates with cognitive symptoms (e.g. Dineen et al., 2009, Sbardella et al., 2013, Meijer et al., 2016a). More recently, there has been evidence of covarying patterns of pathology in white matter tracts. In healthy participants, independent component analysis (ICA) based-approaches have demonstrated patterns of covariance between white matter tracts, thought to reflect shared phylogenetic and functional relationships (Wahl et al., 2010, Li et al., 2012). It can be expected that tracts that share characteristics and/or are part of the same networks are similarly susceptible to pathology. Using ICA on a TBSS skeleton in a sample of secondary progressive MS patients (SPMS), Meijer et al., (2016a) found eighteen components corresponding to WM tracts, which they visually grouped into six different WM classes on the basis of anatomical features. Fractional anisotropy (FA) values within some of these classes correlated with cognitive function, suggesting cognitively-relevant patterns of neurodegeneration (Meijer et al., 2016a). However, given that a voxel-wise approach was used, the data decomposition method used segmented the white matter into components reflecting the major WM tracts of the brain. Subsequent clustering of tracts was then achieved manually. As an extension of this work, it is important to understand whether patterns of pathology are also present across groups of tracts identified by an unbiased data decomposition approach. This is necessary to test whether groups of tracts are similarly susceptible to MS pathology.

The method for assessing WM is also an important consideration. The standard for whole brain WM analysis has long been TBSS, which works by skeletonising the centre of each tract, based on high average FA values, to improve registration of non-homologous brains (Smith et al., 2006). As such TBSS does not reconstruct individual WM tracts, raising concerns about its anatomical accuracy (Bach et al., 2014). Tractography, which fits a diffusion tensor or alternative model at each voxel to trace the fibre orientation through the WM (Mori et al., 1999, Basser et al., 2000, Catani et al., 2002, Jeurissen et al., 2019), provides an alternative for obtaining anatomically accurate WM tracts. While challenging in its own right, technological developments have improved the ease and accuracy of individual, automated tractography (Warrington et al., 2020), and it has been shown that newer tracking algorithms can perform satisfactorily in the presence of MS lesions, and reconstruct even tracts with a high prevalence of lesions (Lipp et al., 2020). This makes tractography a feasible option for segmenting the brain into a large number of functionally meaningful WM units for investigating whether damage to non-lesioned parts of specific tracts can help understand cognitive symptoms in MS.

In the present study we conduct an analysis of WM microstructure diffusion metrics in a large sample of RRMS patients using a tractometry approach (Bells et al., 2011). We use automated individual tractography to reconstruct 40 WM tracts and extract four diffusion metrics from the non-lesioned parts of the tracts. By conducting principal component analysis (PCA) of extracted metrics we can test whether their grouping reflects the known network structure of the brain and covarying patterns of damage across tracts. Exploring this can help us understand the patterns of degeneration in normal appearing tissue in MS.

Thus, the present study aims to: 1) determine if WM tracts can be decomposed into components of shared covariance based on a network or pathology structure; 2) assess the cognitive domains structure present in common neuropsychological test data; 3) explore the relationship between WM tract components and cognitive domains in RRMS.

## 2 Material and methods

### 2.1 Participants

Demographic, clinical and MRI data was collected in one study session from 102 RRMS patients and 27 healthy controls. This cohort has also been investigated and described in previous work (Jandric et al., 2021b). All participants were between 18 and 60 years of age, right-handed and had no contraindications for MR scanning. Patients fulfilled additional eligibility criteria of having no relapses or change to treatment for 3 months prior to the MRI scan, and not having any comorbid neurological or psychiatric disease.

Patients were recruited through the Helen Durham Centre for Neuroinflammation at the University Hospital of Wales and controls from the community. The study was approved by the NHS South-West Ethics and the Cardiff and Vale University Health Board R&D committees. All participants provided written informed consent to participate in the study.

### 2.2 Cognitive assessment

Participants were assessed with the Multiple Sclerosis Functional Composite (MSFC) (Cutter et al., 1999) and the Brief Repeatable Battery of Neuropsychological Tests (BRB-N) (Amato et al., 2006). The BRB-N consists of the following tests: the selective reminding test of verbal memory, which is scored as the sum of words in long term storage (SRT L sum), the sum of words consistently recalled (SRT C sum) and the words recalled after a delay (SRT delayed); the spatial recall test of visual memory, which is scored over three consecutive trials (Spatial1to3) and on a delayed trial (Spatial delayed); the symbol digit modalities test (SDMT) of attention and concentration; the paced auditorial serial addition test of processing speed, with a three second delay (PASAT3) and with a two second delay (PASAT2); and the word list generation test (WLG) of verbal fluency.

### 2.3 MRI acquisition

MRI data were acquired on a 3 T MR scanner (General Electric HDx MRI System, GE Medical Devices, Milwaukee, WI) using an eight channel receive-only head RF coil. A high-resolution 3D T1-weighted sequence was acquired for identification of T1-hypointense MS lesions, segmentation, registration and volumetric measurements (voxel size = 1 mm × 1 mm × 1 mm, TE = 3.0 ms, TR = 7.8 ms, matrix = 256x256x172, FOV = 256 mm × 256 mm, flip angle = 20°). A T2/proton-density (PD)-weighted sequence (voxel size = 0.94 mm × 0.94 mm × 4.5 mm, TE = 9.0/80.6 ms, TR = 3000 ms, FOV = 240 mm × 240 mm, 36 slices, flip angle = 90°) and a fluid-attenuated inversion recovery (FLAIR) sequence (voxel size = 0.86 mm × 0.86 mm × 4.5 mm, TE = 122.3 ms, TR = 9502 ms, FOV = 220 mm × 220 mm, 36 slices, flip angle = 90°) were acquired for identification and segmentation of T2-hyperintense MS lesions. A twice refocused diffusion-weighted spin echo echo-planar (SE-EPI) sequence with 6 volumes with no diffusion weighting and 40 volumes with diffusion gradients applied in uniformly distributed directions was acquired for tractometrics analyses (diffusion directions: Camino 40, b = 1200 s/mm^2^, voxel size = 1.8 mm × 1.8 mm × 2.4 mm, TE = 94.5 ms, TR = 16000 ms, FOV = 230 mm × 230 mm, 57 slices, flip angle = 90°). In addition, a 3D MT sequence (voxel size = 0.94 mm × 0.94 mm × 1.9 mm, TE = 1.8 ms, TR = 26.7 ms, FOV = 240 mm × 240 mm, flip angle = 5°) and mcDESPOT sequence (voxel size = 1.7 mm × 1.7 mm × 1.7 mm, TE = SPGR: 2.1 ms, bSSFP: 1.6 ms, IR-SPGR: 2.1 ms, TR = SPGR: 4.7 ms, bSSFP: 3.2 ms, IR-SPGR: 4.7 ms, FOV = 220 mm × 220 mm, flip angle = SPGR: [3, 4, 5, 6, 7, 8, 9, 13, 18] degrees bSSFP: [10.6, 14.1, 18.5, 23.8, 29.1, 35.3, 45, 60] degrees IR-SPGR: 5°) were acquired to obtain microstructure parameter maps as described in Lipp et al. (2019).

### 2.4 MRI processing

#### 2.4.1 Structural image analysis and lesion marking

Structural 3D T1-weighted images from patients were lesion filled to allow better segmentation and registration of brain tissue. This was done by first segmenting lesions on T2-weighed images using the JIM software (v.6, Xinapse), consulting also the FLAIR and PD-weighted images. Two independent operators performed this segmentation and inter-rated reliability was statistically assessed. Lesion filling was achieved using FSL’s *lesion_filling* function (Battaglini et al., 2012), to estimate intensities from surrounding white matter to ‘fill’ the lesions with. This processed is described in more detail in previous work (Lipp et al., 2019).

Next, lesion filled 3D T1-weighted images were skull-stripped using FSL’s *bet* function (options -m -S -B -f .25 -o -m), then segmented into grey matter (GM), WM and cerebrospinal fluid (CSF) using FSL’s Automated Segmentation Tool (FAST) (Zhang et al., 2001). Intracranial volume (ICV) was calculated with *fslstats* as the number of voxels in skull-stripped T1-weighted images*.* Volumetric measurements normalised for head size, including normalised brain volume (NBV), normalised GM volume (NGMV) and normalised WM volume (NAWM) were quantified from lesion-filled 3D T1-weighted images with FSL’s SIENAX tool (Smith et al., 2002). Lesion volume was calculated from binary lesion masks created as part of lesion marking. The lesion-filled 3D T1-weighted images were non-linearly registered to the Montreal Neurological Institute (MNI) 152 template space using FSL’s FNIRT tool and the warps saved for subsequent analyses.

#### 2.4.2 dMRI analysis: quantification of FA and RD maps

Preprocessing of dMRI data in ExploreDTI (v 4.8.3; Leemans et al., 2009) included motion correction and corrections for eddy current and EPI‐induced geometrical distortions. The latter was achieved by registering each diffusion image to its respective (skull-stripped and downsampled to 1.5 mm) 3D T1 image (Irfanoglu et al., 2012) using Elastix (Klein et al., 2010), with appropriate reorientation of the diffusion‐encoding vectors (Leemans and Jones, 2009). As these data were to be fed into FSL’s Xtract tool, dMRI images were further processed in FSL. The FDT tool was used to fit diffusion tensors and the Bedpostx tool to fit the probabilistic diffusion model (Behrens et al., 2003, Behrens et al., 2007). Fractional anisotropy (FA) and radial diffusivity (RD) maps were normalised to MNI space through the application of the previously obtained warps. FA and RD maps were available for all participants.

### 2.5 MTR and MWF maps

Magnetisation transfer ratio (MTR) and myelin water fraction (MWF) maps were calculated as described in Lipp et al., (2019), which included non-linear co-registration with participants’ T1-weighted images using Elastix (Klein et al., 2010). The warps obtained from nonlinear registration of T1-weighted images to MNI space were then applied to MTR and MWF maps already in T1-space, to achieve registration to MNI space. MTR maps were obtained for all HC and 101 MS patients, and MWF for 25 HC and 95 MS patients. MTR and MWF maps could not be obtained for some participants due to specific absorption rate (SAR) constraints of the mcDESPOT sequence, or due to logistical reasons.

### 2.6 Tractography and tractometry

Bedpostx outputs and T1-weighted to MNI registration warps were fed into FSL’s Xtract tool which uses standardised protocol seeding, exclusion, waypoint and termination masks to perform automated individual tractography to reconstruct 42 WM tracts, then uses the warps to register the outputs to MNI space (Warrington et al., 2020). Automated, individual tractography was chosen over atlas-based tract segmentation approaches to improve the accuracy of tract extraction in individual participants and therefore reduce noise and improve statistical power when extracting diffusion metrics from each tract. All tracts were visually inspected to ensure that they had reconstructed well. In a large proportion of participants, both MS and HC, the fornix failed to reconstruct or was missing portions of the tract. As such, it was not considered for any analyses. The remaining 40 tracts yielded reconstructions in line with their anatomical descriptions and were retained.

Because the protocol masks for defining anatomical start, stop, waypoint and exclusion points of the tract are based on tract atlases, they can lead to extracted tracts having some grey matter. To ensure that tract masks used for our analyses were limited to the WM to be suitable for tractometry analyses, we first thresholded the masked probabilistic tractography outputs at 0.001 and then masked the output further with the respective WM mask from the segmented T1-weighted scan. These tracts were binarised and all voxels marked as lesions were removed to get a mask of only the non-lesioned part of the tract. The proportion of each tract affected by lesions in each participant was calculated by counting the lesion voxels in each tract relative to the voxels of the whole tract, averaged over all 102 participants for each tract.

To obtain FA, RD, MTR and MWF metrics from each reconstructed tract, each metric was averaged across all voxels in the non-lesioned tract masks. Distributions of each metric in each tract were assessed through histogram inspection in MATLAB (v R2020a). The majority of the FA, RD, MTR and MWF tract maps had distributions deviating from normality so median values were extracted.

#### 2.6.1 Metrics dimensionality reduction

The four WM microstructure metrics were extracted from each tract and decomposed into one metric using principal component analysis. This dimensionality reduction analysis was performed on the FA, RD, MWF and MTR metrics in RStudio v 1.4.1103 using the *principal* function (RStudio Team, 2020). Mean values were imputed for the missing MTR and MWF values and a dataset comprising of 4 WM metrics × 40 WM tracts × 27 or 102 participants (for HC and MS, respectively) was created. The four metrics were reduced to a lower dimensionality that explains the maximum amount of variance in the data through a PCA performed across participants and tracts, as described by Chamberland et al., (2019). First, a correlation matrix of Pearson’s *r* was calculated to determine feasibility of a PCA based on high correlations and tested with Bartlett’s test of sphericity to ensure a significant difference from an identity matrix. The metric principal component for further analyses was chosen on the basis of an inspection of the scree plot (Cattell, 1966) and eigenvalues > 1. A metric component score for the first extracted principal component, explaining most variance, was calculated for each tract and participant.

#### 2.6.2 Principal component analysis of WM tract covariance

To assess whether patterns of shared covariance exist across the WM, an additional PCA, following the same process, was performed in HC and MS, respectively. For this PCA, the metric component score of the first extracted component was used as the WM microstructure metric for each tract.

#### 2.6.3 Regression of sources of heterogeneity in data

To identify the sources of variance in a tract component (TC) resulting from this PCA, its component scores were correlated with a number of demographic and anatomical variables: age, sex, years of education, ICV, lesion volume, NBV, NGMV and NWMV. Multiple regressions were performed to identify which of these variables explained most variance of the TC. First, all demographic and anatomical were inputted into a correlation matrix to assess the degree of multicollinearity. As there was high correlation between NBV, NGMV and NWMV, only NBV was included in the model, along with age, sex, education ICV and lesion volume. The demographic and anatomical variables that came out as the strongest predictors in the regression analysis were regressed out of the raw data and the metric dimensionality reduction and PCA of WM tract covariance steps were performed again. The aim of this was to take into account strong general effects of certain variables on all considered white matter tracts. In particular, the effects of lesion volume were regressed out to allow us to explore whether microstructural white matter variance is shared similarly across some tracts compared to others, potentially demonstrating shared susceptibility to non-lesional pathology. A Varimax rotation was applied to the first four principal components, based on eigenvalues > 1 and proportion variance explained, to improve interpretability.

#### 2.6.4 Cognitive test principal component analysis

Finally, we aimed to find the cognitive domain structure in this dataset. As for the metric and tract PCAs, a correlation matrix was constructed based on the scores on each of the BRB-N tests, and on the basis of confirmed correlations between tests and a significant Bartlett’s test, a PCA was performed to decompose the battery tests into cognitive domains. Principal components were extracted on the basis of scree plots, eigenvalues and variance explained. A Varimax rotation was applied for interpretability. To understand what influences cognitive function, the resulting rotated cognitive components (CCs) were correlated with the tract components and all demographic and anatomical variables, after checking multicollinearity among predictors. NBV, NGMV and NWMV correlated highly so the variables included were age, sex, education, ICV, lesion volume and NBV. Multiple regression analyses were performed to determine the relationship between WM tract microstructure and cognitive domains, while controlling for variables which may also influence cognitive function.

### 2.7 Statistical analyses

All analyses were performed in RStudio v 1.4.1103 (RStudio Team, 2020) with the exception of analyses of demographic and clinical variables, which were analysed in SPSS version 23.0 (IBM Corp., 2015). All variables were tested for normality through visual inspection of histograms and Q-Q plots and application of Kolmogorov-Smirnov tests. A significance threshold of *p* < 0.05 was applied unless otherwise indicated. For all multiple regression models, the adjusted R-squared is reported, to adjust for the number of predictors in the model. For individual predictors in the model, the reported coefficients are standardised beta coefficients calculated with the *lm.beta* function in R.

## 3 Results

### 3.1 Participant characteristics

Demographic and clinical characteristics of the sample are presented in Table 1. Overall, patients were older and less educated than healthy controls, had lower NBV and NGMV, poorer upper and lower limb function, and performed worse on all cognitive tests except the word list generation test assessing verbal fluency.

**Table 1:** Demographic, clinical and neuropsychological characteristics.

	HC (n = 27)	RRMS (n = 102)	Inferential test results
Age, yr (median, range)	37.00 (23–59)	45.00 (18–60)	*U* = 958.00, *p* = 0.015
Male/female, n	12/15	33/69	*χ*^2^(1) = 1.37, *p* = 0.241
Education years (median, range)	19.00 (12–30)	15.00 (10–30)	*U* = 613.50, *p* < 0.001
Mean disease duration, yr (median, range)	N/A	12.24 (1–39)	N/A
Timed 25 Foot Walk Test (median, range)	4.35 (3.2–5.4)	5.25 (3.6–26.8)	*U* = 572.50, *p* < 0.001
9-Hole Peg Test (median, range)	18.65 (15.35–23.00)	21.75 (16.35–59.50)	*U* = 537.50, *p* < 0.001
SRT L sum (median, range)	0.00 (-1.26–1.37)	−0.54 (-4.72–1.47)	*U* = 914.00, *p* = 0.009
SRT C sum (mean, SD)	0.00 (1.00)	−0.88 (1.22)	*t*(49.06) = 3.86, *p* < 0.001
SRT delayed (median, range)	0.06 (-2.13–1.16)	−0.49 (-4.31–1.15)	*U* = 881.00, *p* = 0.004
Spatial1to3	0.00 (1.00)	−0.74 (1.20)	*t*(47.76) = 3.29, *p* = 0.002
Spatial delayed	0.11 (-2.45–1.14)	−0.91 (-2.96–1.14)	*U* = 794.00, *p* = 0.001
SDMT	0.00 (1.00)	−0.88 (0.98)	*t*(40.14) = 4.09, *p* < 0.001
PASAT3 (median, range)	−0.03 (-2.61–1.26)	−1.32 (-8.26–1.42)	*U* = 692.00, *p* < 0.001
PASAT2	0.17 (-1.71–2.29)	−0.77 (-4.45–2.41)	*U* = 768.00, *p* < 0.001
WLG	0.00 (1.00)	−0.24 (0.88)	*t*(37.48) = 1.14, *p* = 0.263
Normalised brain volume, L (mean, SD)	1.56 (0.07)	1.51 (0.08)	*t*(41.94) = 3.33, *p* = 0.002
Normalised grey matter volume, L (median, range)	0.81 (0.72–0.89)	0.77 (0.61–0.89)	*U* = 755.00, *p* < 0.001
Normalised white matter volume, L (median, range)	0.76 (0.68–0.81)	0.74 (0.66–0.83)	*t*(40.43) = 1.56, *p* = 0.127

Independent samples *t*-tests were used for comparisons of variables with a normal distribution. Mann-Whitney U tests were used for variables which were not normally distributed. Sex, being a categorial variable, was tested with the chi-squared test. Cognitive test scores are reported as Z-scores.

Abbreviations: HC = healthy controls; PASAT2 = paced auditory serial addition test 2 s delay; PASAT3 = paced auditory serial addition test 3 s delay; RRMS = relapsing remitting multiple sclerosis; SD = standard deviation; SDMT = symbol digit modalities test; Spatial1to3 = spatial recall test average score over three trials; Spatial delayed = spatial recall test score at the delayed trial; SRT delayed = serial recall test scores at the delayed trial; SRT L sum = serial recall test long term storage sum of scores; SRT L sum = serial recall test consistent recall sum of scores; WLG = word list generation test.

### 3.2 Metric dimensionality reduction

The results in the following sections are for the MS group unless otherwise indicated. Bartlett’s test of sphericity was significant (χ^2^(6) = 155.85, *p* < 0.001) indicating the suitability of performing a PCA. Based on scree plot inspection and eigenvalues > 1, only the first principal component, which explained 60% of variance, was extracted. The component loadings were 0.92 for FA, −0.87 for RD, 0.88 for MWF and 0.15 for MTR, indicating that the main contributors to the component were FA, RD and MWF.

### 3.3 Principal components of WM tract covariance

**Fig. 1:**
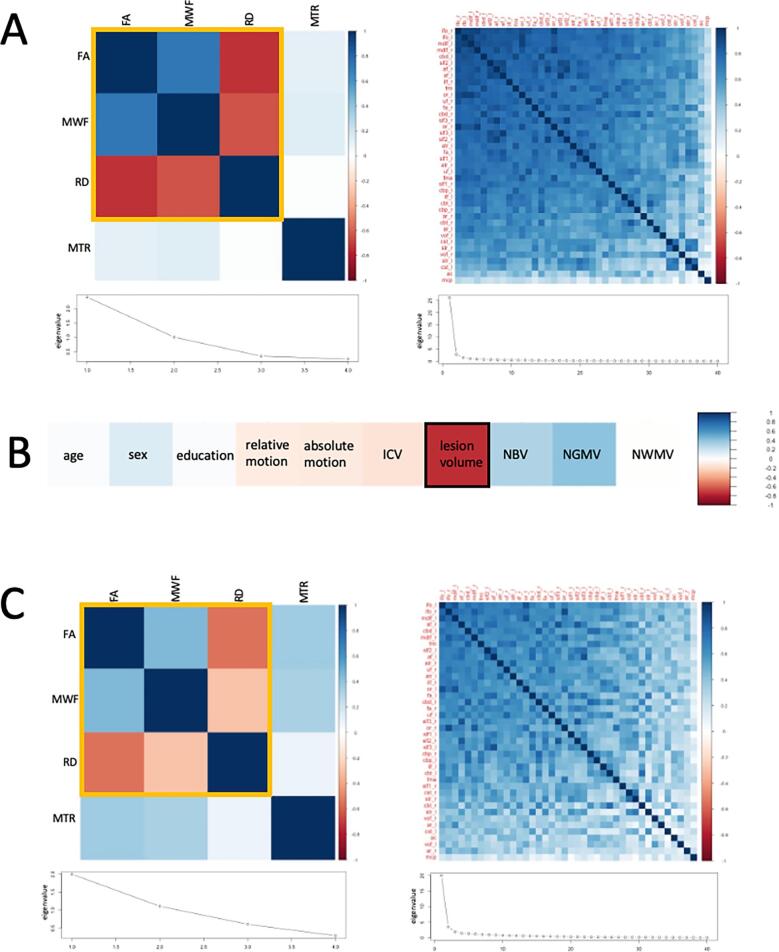
Metric and tract principal component analysis in healthy controls. Fig. 1A shows the correlation matrices and scree plots for the PCA ran on the four white matter microstructural metrics (left) and the white matter tracts based on the first component from the metric PCA (right). Those metrics marked with a yellow line load most on principal component 1. All tracts loaded positively on tract principal component 1. Fig. 1B shows correlations between rotated tract principal component 1 (TC1) and demographic and anatomical variables. Variable marked with a black line, lesion volume, was a significant predictor of the principal tract component from Fig. 1A in multiple linear regression models. Fig. 1C shows the correlation matrices and scree plots for metric and tract PCAs after lesion volume was regressed out. Abbreviations: FA = fractional anisotropy; RD = radial diffusivity; MWF = myelin water fraction; MTR = magnetisation transfer ratio; ICV = intracranial volume; NBV = normalised brain volume; NGMV = normalised grey matter volume; NWMV = normalised white matter volume. (For interpretation of the references to colour in this figure legend, the reader is referred to the web version of this article.)

In MS patients, a correlation matrix of all WM tracts was shown to be significantly different from an identity matrix using Bartlett’s test of sphericity (χ^2^(780) = 5803.14, *p* < 0.001), indicating the suitability of performing a PCA to assess the covariance structure of WM tracts (see Fig. 1A for the metric and tract correlation matrices and scree plots). The scree plot showed one strong principal component (65% variance explained), but three additional components had eigenvalues > 1 (7%, 4% and 3% variance explained, respectively). A Varimax rotation was therefore applied to these first four principal components to improve interpretability. After rotation all tracts still loaded positively on TC1, demonstrating a great degree of shared variable between white matter tracts.

In MS patients TC1 correlated most strongly with lesion volume (*r* = -0.73), NGMV (*r* = 0.41), and NBV (*r* = 0.31) (see Fig. 1B). A multiple linear regression model showed that the variance of TC1 was best explained by lesion volume (ß = −0.74, *p* < 0.001) in a model explaining 54% of variance (R^2^ = 0.54, *F*(6, 95) = 20.58, *p* < 0.001). See Table 2 for full model statistics.

**Table 2:** Predictors of WM tract covariance and cognitive domains.

Dependent variable	Predictors	Model statistics
Unrotated tract component 1	Age: ß = 0.03, *p* = 0.727	R^2^ = 0.54, *F*(6, 95) = 20.58, *p* < 0.001
	Sex: ß = 0.13, *p* = 0.186	
	Education: ß = −0.03, *p* = 0.690	
	ICV: ß = −0.03, *p* = 0.721	
	*Lesion volume: ß = −0.74, p < 0.001*	
	NBV: ß = 0.01, *p* = 0.452	
CC1: Verbal cognition	*TC1: ß = 0.30, p = 0.009*	R^2^ = 0.17, *F*(10, 91) = 3.04, *p* < 0.001
	TC2: ß = −0.07, *p* = 0.444	
	TC3: ß = 0.14, *p* = 0.120	
	TC4: ß = 0.08, *p* = 0.379	
	Age: ß = −0.01, *p* = 0.957	
	*Sex: ß = 0.35, p = 0.010*	
	Education: ß = 0.06, *p* = 0.558	
	ICV: ß = 0.12, *p* = 0.365	
	*Lesion volume: ß = −0.22, p = 0.049*	
	*NBV: ß = −0.32, p = 0.028*	
CC2: Visuospatial cognition	TC1: ß = −0.01, p = 0.938	R^2^ = 0.05, *F*(10, 91) = 1.50, *p* = 0.153
	TC2: ß = −0.13, p = 0.191	
	TC3: ß = 0.08, p = 0.442	
	TC4: ß = −0.11, p = 0.290	
	*Age: ß = −0.32, p = 0.009*	
	Sex: ß = 0.10, p = 0.482	
	Education: ß = 0.09, p = 0.358	
	ICV: ß = 0.01, p = 0.963	
	Lesion volume: ß = −0.04, p = 0.708	
	NBV: ß = −0.04, p = 0.800	
CC3: Information processing	TC1: ß = 0.06, p = 0.628	R^2^ = 0.06, *F*(10, 91) = 1.65, *p* = 0.106
	TC2: ß = 0.12, p = 0.236	
	*TC3: ß = 0.24, p = 0.017*	
	TC4: ß = −0.16, p = 0.101	
	Age: ß = 0.18, p = 0.127	
	Sex: ß = −0.14, p = 0.318	
	Education: ß = −0.02, p = 0.839	
	ICV: ß = −0.10, p = 0.493	
	Lesion volume: ß = 0.04, p = 0.739	
	NBV: ß = 0.20, p = 0.197	
CC4: Executive function	TC1: ß = −0.02, p = 0.870	R^2^ = 0.18, *F*(10, 91) = 3.19, *p* = 0.002
	TC2: ß = 0.05, p = 0.604	
	TC3: ß = 0.15, p = 0.101	
	TC4: ß = 0.03, p = 0.747	
	*Age: ß = −0.27, p = 0.016*	
	Sex: ß = −0.03, p = 0.851	
	Education: ß = 0.07, p = 0.443	
	ICV: ß = −0.02, p = 0.872	
	*Lesion volume: ß = −0.23, p = 0.035*	
	NBV: ß = 0.13, p = 0.352	

Significant predictors are presented in italics. Significance threshold *p* < 0.05 applied unless otherwise indicated. Abbreviations: CC = cognitive component, NBV = normalised brain volume, NWMV = normalised white matter volume, TC = tract component, WM = white matter.

After regressing out lesion volume, correlations matrices for WM metrics and tracts, respectively, showed somewhat weaker correlations but still passed Bartlett’s test of sphericity (χ^2^(6) = 90.01, *p* < 0.001 for metrics, χ^2^(780) = 4347.86, *p* < 0.001 for tracts), and yielded the same PCA structure (see Fig. 1C), indicating that most tracts still load positively onto a single component. After a component rotation of the four tracts that explained most of the variance (after rotation:79% cumulative variance; 30%, 25%, 21%, 0.03% for TCs 1–4, respectively) most tracts still loaded positively on the first tract component, especially large WM tracts like the optic radiations, middle longitudinal fasciuli, forceps major, inferior fronto-occipital fasciculi, vertical occipital fasciculi and acoustic radiations. Similarly, the tracts which loaded most highly on TC2 were large tracts connecting distal areas of the brain, including the superior thalamic radiations, corticospinal tracts, frontal aslants, superior longitudinal fasiculi and the arcuate fasciculi. TC3 in contrast consisted mainly of shorter tracts, including sub-sections of the cingulum, the anterior commissure, forceps minor and uncinate fasciuli. Only the middle cerebellar penduncle loaded highly on TC4. The principal component analysis screeplot showing a single dominant component and the high tract loadings of all tracts onto the first of the four rotated components demonstrates a high covariance between all tracts investigated. Please see Table 3 for full details of tract loadings on the four components.

**Table 3:** Tract loadings on each component derived from the tract PCA, after regressing out significant predictors of tract variance and applying Varimax rotation.

TC1		TC2		TC3		TC4	
Tract	Loading	Tract	Loading	Tract	Loading	Tract	Loading
or_l	0.77	str_l	0.89	cbp_r	0.77	mcp	0.84
mdlf_l	0.75	cst_l	0.86	ac	0.73	vof_r	0.26
or_r	0.75	str_r	0.82	cbd_r	0.72	ac	0.25
fma	0.75	cst_r	0.78	cbt_r	0.70	fma	0.23
mdlf_r	0.74	fa_l	0.73	cbt_l	0.67	ar_r	0.23
ifo_r	0.72	fa_r	0.68	cbp_l	0.67	or_r	0.23
vof_l	0.70	af_l	0.66	fmi	0.65	cst_r	0.23
ar_l	0.70	slf1_l	0.65	uf_l	0.64	atr_r	0.21
vof_r	0.69	slf3_l	0.65	cbd_l	0.63	atr_l	0.18
ar_r	0.68	af_r	0.63	uf_r	0.60	mdlf_r	0.16
ifo_l	0.67	slf2_r	0.62	atr_l	0.59	ifo_r	0.14
ilf_r	0.67	slf2_l	0.59	atr_r	0.58	ilf_l	0.12
slf2_l	0.62	atr_l	0.59	ilf_l	0.55	fmi	0.12
af_r	0.62	slf3_r	0.59	ifo_l	0.50	af_r	0.09
slf1_r	0.61	atr_r	0.56	ilf_r	0.46	or_l	0.09
slf3_r	0.60	mdlf_r	0.47	mdlf_l	0.44	slf3_r	0.08
ilf_l	0.58	slf1_r	0.44	ifo_r	0.43	cbt_r	0.08
slf2_r	0.58	ifo_l	0.44	fa_l	0.39	ilf_r	0.07
af_l	0.56	ifo_r	0.43	slf1_l	0.39	str_r	0.06
cbd_l	0.56	cbd_l	0.42	fma	0.38	uf_r	0.04
uf_r	0.55	fmi	0.41	slf2_l	0.37	slf2_r	0.03
cbt_l	0.54	mdlf_l	0.40	or_l	0.37	ifo_l	0.02
slf3_l	0.53	or_r	0.40	ar_l	0.37	vof_l	0.02
fa_r	0.52	ilf_r	0.39	af_l	0.34	str_l	0.02
fmi	0.49	uf_r	0.37	mdlf_r	0.33	cbd_r	0.02
cbd_r	0.47	uf_l	0.37	fa_r	0.33	cbd_l	0.01
uf_l	0.45	cbd_r	0.35	vof_l	0.33	cbt_l	0.00
slf1_l	0.44	or_l	0.35	slf1_r	0.32	mdlf_l	0.00
cbt_r	0.44	ar_r	0.32	slf3_l	0.32	slf3_l	−0.03
cbp_l	0.42	cbp_l	0.31	af_r	0.31	cst_l	−0.03
fa_l	0.38	cbp_r	0.27	slf3_r	0.30	slf1_r	−0.03
atr_r	0.31	ilf_l	0.21	or_r	0.29	af_l	−0.04
atr_l	0.31	fma	0.21	slf2_r	0.28	cbp_l	−0.04
cbp_r	0.31	vof_r	0.15	vof_r	0.21	fa_r	−0.04
cst_r	0.24	ar_l	0.14	ar_r	0.17	slf2_l	−0.05
mcp	0.22	cbt_l	0.13	str_r	0.16	cbp_r	−0.05
str_r	0.19	vof_l	0.11	cst_r	0.14	slf1_l	−0.06
str_l	0.14	cbt_r	0.11	mcp	0.13	uf_l	−0.07
cst_l	0.08	ac	0.04	cst_l	0.09	fa_l	−0.12

Abbreviations: ac = anterior commissure; af = arcuate fasciculus; ar = acoustic radiation; atr = anterior thalamic radiation; cbd = cingulum subsection, dorsal; cbp = cingulum subsection, peri-genual; cbt = cingulum subsection, temporal; cst = corticospinal tracr; fa = frontal aslant; fma = forceps major; fmi = forceps minor; ifo = inferior fronto-occipital fasciculus; ilf = inferior longitudinal fasciculus; mcp = middle cerebellar peduncle; mdlf = middle longitudinal fasciculus; or = optic radiation; slf1-3 = superior longitudinal fasciculus 1–3; str = superior thalamic radiation; uf = uncinate fasciculus; vof = vertical occipital fasciulus. Left and right hemisphere tracts are denoted with _l and _r, respectively.

A supplementary analysis was performed to repeat all analysis steps performed in MS patients also in healthy controls. This was done as a control of the tract structure that emerged from the PCA, to understand if the finding of one strong tract component in MS was due to the disease (if the component structure is substantially different from controls) or likely to reflect some general aspect of WM microstructure (if the component structure is similar in controls). The same tract component structure that was found in MS patients was also found in MS, albeit with a slight different in which tracts loaded most likely onto the first tract component. All results from healthy controls are presented in Appendix 1.

To assess the influence of lesions on the present methodological approach, a supplementary analysis was performed in which voxels marked as lesions were not masked out of tract masks before WM metrics were extracted. All other analysis steps were not changed. Including lesioned tissue in the tract when extracting WM metrics did not substantially alter the results, one main tract component was still found. Please see Appendix 2 for results.

### 3.4 Cognitive domains

A correlation matrix of cognitive test scores showed a large number of moderate to high correlations and was significantly different from an identity matrix as assessed by Bartlett’s test of sphericity (χ^2^(36) = 558.62, *p* < 0.001), indicating a likely domain structure of cognition and confirming suitability for a PCA. Based on eigenvalues of at or near 1 and proportion variance explained, four components explaining 85% of variance were extracted. After a Varimax rotation a clear component structure emerged whereby cognitive component (CC) 1 reflects verbal cognition and CC2 visuospatial cognition, while CCs 3 and 4 reflect information processing speed and executive function, respectively. The component weights for rotated cognitive components (CCs) were as follows: Serial Recall Test Consistent recall (0.87), Serial Recall Test Long term storage recall (0.82), Word List Generation Test (0.80) and Serial Recall Test delayed recall (0.73) for CC1; Spatial Recall Test over three trials (0.90) and Spatial Recall Test delayed recall (0.93) for CC2; Paced Auditory Serial Addition Test 3 s delay (0.88) and Paced Auditory Serial Addition Test 2 s delay (0.89) for CC3; and Symbol Digit Modalities Test (0.84) for CC4, see Table 4.

**Table 4:** Cognitive component weights in MS patients.

	Cognitive RC1	Cognitive RC2	Cognitive RC3	Cognitive RC4
SRT L sum	**0.82**	0.16	0.10	0.36
SRT C sum	**0.87**	0.18	0.21	0.24
SRT delayed	**0.73**	0.25	0.29	0.37
Spatial1to3	0.15	**0.90**	0.23	0.06
Spatial delayed	0.10	**0.93**	0.01	0.15
SDMT	0.20	0.17	0.27	**0.84**
PASAT3	0.17	0.18	**0.88**	0.20
PASAT2	0.25	−0.06	**0.89**	0.11
WLG	**0.80**	−0.05	0.17	−0.29

Abbreviations: PASAT2 = paced auditory serial addition test 2 s delay; PASAT3 = paced auditory serial addition test 3 s delay; SDMT = symbol digit modalities test; Spatial1to3 = spatial recall test average score over three trials; Spatial delayed = spatial recall test score at the delayed trial; SRT delayed = serial recall test scores at the delayed trial; SRT L sum = serial recall test long term storage sum of scores; SRT C sum = serial recall test consistent recall sum of scores; WLG = word list generation test.

### 3.5 Predictive ability of tract components, demographic and anatomical variables on cognition domains

In MS, the ability of tract components and demographic and MRI variables to explain variance of cognitive domains was assessed by including them together in regression models. Together, these variables explained 17% variance of cognitive domains (see Table 2). The first cognitive component, CC1, was best predicted by TC1 (ß = 0.30, *p* = 0.009), sex (ß = 0.35, *p* = 0.010), lesion volume (ß = −0.22, *p* = 0.049) and NBV (ß = −0.32, *p* = 0.028), in a model explaining 17% of variance (R^2^ = 0.17, F(10, 91) = 3.04, *p* < 0.001). The final cognitive component, CC4, was best predicted by age (ß = −0.27, *p* = 0.016) and lesion volume (ß = −0.23, *p* = 0.035), in a model explaining 18% of the variance (R^2^ = 0.18, F(10, 91) = 3.19, *p* = 0.002). For cognitive components 2 and 3, the regression models were not significant. Please see Table 2 for full statistical results.

We conducted a supplementary set of analyses to understand if using WM tract components reflects something additional to what a simple global FA measure would. We first correlated mean FA from TBSS to TC1 and found a weak to moderate strength correlation (*r* = 0.32, *p* = 0.001). When regression analyses were repeated with the addition of this mean FA metric as a model predictor no additional variance in cognitive domains was found. We also conducted univariate correlations between all cognitive domains and demographic and anatomical variables to understand the relationship between individual variables and cognition better and found several significant correlations. Full results for these supplementary analyses are presented in Appendix 3.

## 4 Discussion

In this study we combined PCA with tractometry to determine whether cognitive performance in people with MS relates to one or many patterns of white matter tract pathology. A decomposition approach of microstructure metrics from WM tracts showed a high degree of covariance across most tracts, indicating a global WM structure rather than shared relationships among some tracts over others due to functional patterns or susceptibility to pathology. This global WM microstructure component was largely explained by lesion volume, but retained largely a single covariance pattern even after this factor was regressed out. Cognitive domains were only modestly explained by WM microstructure components and other anatomical and demographic variables. These findings do not support the suggestion of shared susceptibility to MS pathology among WM tracts as a brain correlate of cognitive impairment in MS. However, the effect of the methods used on these results must be considered, and is discussed in the following sections.

### 4.1 Metric dimensionality reduction

Traditionally FA is used in MS studies of cognition, but FA has been shown to be susceptible to many factors, including myelination, axonal density and orientational dispersion of fibre populations in a voxel (Beaulieu, 2014, De Santis et al., 2014, Lazari and Lipp, 2021). A dimensionality reduction approach of multimodal WM metrics has been shown to overcome the problem of multiple comparisons of data containing overlapping information while maintaining good sensitivity of WM microstructure (Chamberland et al., 2019, Geeraert et al., 2020, Bosticardo et al., 2021).

Our composite WM metric consisted mainly of FA, RD and MWF, and to a much lower degree, MTR. This finding is consistent with a previous report of poor correlations between MTR and FA, RD and MWF in normal appearing white matter (Lipp et al., 2019). MTR has shown particular sensitivity to lesioned tissue (Lipp et al., 2019, Moccia et al., 2020) and in the present study lesions were masked out to obtained normal appearing white matter only. It is therefore possible that extracted MTR values had limited variance across tracts and/or patients.

### 4.2 WM microstructure organisation

Given the prior evidence showing connectivity changes associated with cognition in MS (reviewed in Chard et al., 2021, Jandric et al., 2021a, Jandric et al., 2021b*a*) and the possibility that they are driven by WM degeneration (Catani and ffytche, 2005, Dineen et al., 2009, Schoonheim et al., 2015), we aimed to assess whether WM tracts can be decomposed based on shared pathological or other features, and whether the resulting components reflect known functional network structures.

Our results provide limited evidence of separate covariance structures of WM tracts in MS patients. A single dominant component consisting of all tracts was found in both people with MS and healthy controls, although with somewhat different tract loadings. In MS the main component was largely explained by lesion volume. Even though lesions were masked out of each tract and only non-lesioned tissue was included in the analyses, inflammatory activity in lesions is known to have an effect on surrounding tissue and Wallerian and retrograde degeneration is known to occur in remote areas from the lesions (Trapp et al., 1998, Bitsch et al., 2000, Trapp and Stys, 2009). After regressing out this predictor, the tract pattern covariance was still shared between all tracts (i.e. no separate patterns of pathology emerged). This suggest that although lesion effects explained about 50% of variance in this principal tract component, they were not the main source of variance shared between tracts that resulted in the analysis yielding only one strong component. Rather, it is likely that this component reflects some global aspect of white matter microstructure.

There are a number of possible reasons for why pathology patterns associated with functional networks may not have emerged. First, white matter may not show a strong network structure or patterns of covarying pathology. We also found only one dominant tract component in healthy controls, and thus no evidence of a network structure in the white matter (see Appendix 1). This is consistent with a previous study in elderly healthy controls that found a single general factor explaining almost half of variance across all eight tracts assessed (Penke et al., 2010). Studies which do report patterns of WM pathology have grouped tracts into classes manually, rather than statistically based on shared features (Li et al., 2012, Meijer et al., 2016a). However, despite manual grouping, each class determined by Meijer et al., (2016a) did show that both FA values and component loadings within a class were associated with cognition, suggesting possible shared damage within a class. Thus it is important to investigate this potential shared susceptibility to pathology among tracts further.

A second possible reason for the lack of several principal components is that patterns of WM pathology may only emerge at later stages of the disease. While network changes measured by rs-fMRI are common in RRMS and occur even in clinically isolated syndrome (CIS, reviewed in Jandric et al., 2021a, Jandric et al., 2021b), they have been shown to be more pronounced in progressive MS (Meijer et al., 2018, Rocca et al., 2018), and the severity of brain pathology is generally related to disease duration (Reynolds et al., 2011). It is therefore feasible that if there is shared susceptibility to MS pathology in the WM, like in the grey matter, it comes more pronounced as the disease advances. This would need to be tested in longitudinal studies or large cross-sectional studies with both RRMS and SPMS samples.

In addition, patterns of pathology may only become apparent when looking at regions within tracts and not, as assessed in this study, across whole tracts. It is known that many major tracts support several separate functions, for example the interior fronto-occipital fasciculus is involved in cognition and sensorimotor functions as well as other behaviours (Sarubbo et al., 2013). Indeed, Li et al., (2012) found different FA covariance patterns for different segments of the corpus callosum. In support of this point, a recent study found that WM tract metrics of volume and microstructural integrity from specific section of specific tracts, including subsections of the corpus callosum, superior longitudinal fasciculus and the striato-prefrontal and striato-parietal pathways, better predict cognitive test performance than global tractography and lesion measures, and also better than whole tract measures (Winter et al., 2021). This lack of granularity in our data may therefore account for the weak component structure that emerged after rotation. Further studies comparing regional ICA and PCA approaches can help to determine the extent to which each of these factors is at play.

### 4.3 Relationships between WM microstructure and cognition

Applying the same data decomposition approach to neuropsychological test data we identified four cognitive domains: verbal cognition, visuospatial cognition, information processing speed and executive function, consistent with the known domain structure of the BRB-N (Sepulcre et al., 2006, De Meo et al., 2021).

We found that the first and main tract component was related to specific cognitive domains, but overall these associations were not strong. This component, together with sex, lesion volume and normalised brain volume, explained less than 20% of the variance of the verbal cognitive domain. This tract component is made up of most of the tracts investigated, but those which load most highly are long association tracts which connect most of the brain. Interesting, tracts which connect the occipital cortex to the rest of the brain load highly onto this tract component. It may seem as an unexpected finding that tracts associated with visual function predict a cognitive domain without a visual element, but it is important to consider that we found all tracts to correlate highly with each other, so this correlation between the tract component and cognition is not specific to visual tracts. Nevertheless, damage to the occipital cortex, including atrophy and functional connectivity abnormalities, (in line with known pathology within the optic nerve, i.e. optic neuritis) is commonly reported in MS (Pagani et al., 2005, Calabrese et al., 2007, Tona et al., 2014), so the present finding may reflect a non-cognitively specific marker of MS pathology, albeit weakly, as the tract component only explained a small proportion of this cognitive domain. Lesion volume and atrophy measures were also modest predictors of cognitive domain variance, confirming the clinical-radiological paradox and the need for more advanced brain pathology measures in the study of cognitive impairment in MS.

The modest relationship between test performance on the different cognitive domains and WM tract components contrasts with previous evidence linking WM microstructure in MS to cognitive function (Dineen et al., 2009, Inglese and Bester, 2010, Hulst et al., 2013, Sbardella et al., 2013, Llufriu et al., 2014, Meijer et al., 2016b). Given that all tracts loaded onto the first tract component, and it can therefore be interpreted as reflecting some global aspect of white matter microstructure, a stronger relationship with cognition may have been expected given prior research. This may be due to our use of whole tract measures. There is evidence to suggest that spatial topography is important for cognitive deficits and that some tracts in particular are involved in supporting cognitive function. Most of the early diffusion studies of cognition in MS report correlations between cognitive performance and diffusion metrics in specific regions of tracts, despite analyses being conducted over a whole brain WM skeleton. Those which are commonly reported across the literature are the corpus callosum, cingulum and forceps major and minor (Dineen et al., 2009, Sbardella et al., 2013, Llufriu et al., 2014). In addition, in another study of the sample investigated in the present study, WM metric differences between cognitively impaired and non-impaired patients were found mainly in the corpus callosum and cingulum (Jandric et al., 2021b).

This possibility of spatial specificity has not been formally established through a meta-analysis to date and is therefore speculative. However, it is supported by recent graph theory studies which have found associations between structural characteristics of predefined networks and cognitive function rather than across the whole connectome (Llufriu et al., 2017, Llufriu et al., 2019, Koubiyr et al., 2019, Has Silemek et al., 2020). The third tract component identified in this study had had loadings from sections of the cingulum and forceps minor, yet did not explain a great deal of variance of cognitive domains. However, this tract component also consisted of a large number of other tracts with high loadings, so is non-specific to the cingulum and corpus callosum. Future work should focus on establishing if certain WM tracts in particular, e.g. those connecting key hub regions of the brain, are more important for cognitive function and more susceptible to pathology. For these purposes, graph theory may be informative in future work. While this approach is a promising tool for studying cognitive impairment in MS, it relies on many decisions regarding how the network is constructed (Yeh et al., 2020). The approach described in this paper was an attempt to identify such networks in a data-driven way. By looking at well defined specific white matter tracts, we ensured anatomical interpretability and plausibility. Since we could only identify one main component across these tracts, a more fine-grained way of looking at connectivity between grey matter regions may be necessary for future attempts on data-driven network definition.

### 4.4 Limitations

Our study is not without limitations. Few previous studies have used data decomposition approaches to WM metrics in order to separate sources of signals, such as independent component analysis (ICA) (Li et al., 2012, Meijer et al., 2016a). In both studies the WM skeleton fed into the ICA returned components which reflected individual tracts or sub-sections of tracts. This is a complicated method to obtain individual tract masks, which in our study was achieved by tractography as a first analysis step. Grouping of tracts in both previous studies was done manually, which is an arbitrary approach to tract clustering, introducing the risk of bias. In contrast, we used PCA in an exploratory analysis to identify patterns of shared variance. This minimises the risk of bias and may also better reflect normal variation in white matter structure. By comparing dominant components between control and patients we were able to evaluate whether such structures are to be expected. However, the possibility should be considered that tract components are not actually orthogonal, perhaps due to the known multifunctional nature of WM tracts, and independent component analysis would in this case have been a more suitable approach. We could also have used factor analytic techniques, but PCA has been shown to produce very similar results to factor analysis when the communalities of variables investigated are greater than 0.7, which was the case in this study (Guadagnoli and Velicer, 1988).

Finally, graph theory connectomics is an alternative approach to investigating the network structure of white matter. Due to the exploratory nature of the present study, a simpler method, which is able to capture anatomical accuracy of white matter tracts well, was employed for an initial investigation of shared relationships between tracts. However, it is clear that our results reflect some element of shared variance across all tracts investigated, which was stronger than any shared susceptibility to other factors, like involvement in different brain networks, which graph theory approaches have been shown to be able to tap into (Sporns and Betzel, 2016). In summary, this nascent research area requires further detailed work to determine the optimal analysis strategy to identify patterns of white matter pathology. In doing so they can help to understand whether there are networks that are susceptible to MS disease and how these might change over time.

### 4.5 Conclusions and future directions

In this study we explored whether non-lesioned portions of white matter tracts share variance among some tracts over others, which could indicate shared susceptibility to non-lesion pathology in MS. We did not find evidence of this, instead, our results revealed one strong component reflecting variance from all tracts, suggesting that our chosen method showed some shared aspect of white matter microstructure, which may be influenced by pathological processes in MS. This pattern of WM tract variance showed a modest relationship with cognitive function.

The study raises several questions about whether, or not, there is a structure to the pathological changes underlying cognitive impairment in MS, and to what extent it is influenced by the methods used. An important aim for future research is to compare different approaches to investigating white matter connectivity to understand how best to probe shared vulnerability to MS pathology among tracts. By doing so we can develop a greater understanding of why spatially heterogeneous damage may lead to similar impairments to affect the lives of people with MS.

## Funding

This work was funded by a research grant of the MS Society UK and a Medical Research Council Doctoral Training Partnership grant (MR/N013751/11).

### CRediT authorship contribution statement

**Danka Jandric:** Conceptualization, Methodology, Software, Validation, Formal analysis, Writing – original draft, Visualization. **Geoff J.M. Parker:** Validation, Supervision, Writing – review & editing. **Hamied Haroon:** Validation, Supervision, Writing – review & editing. **Valentina Tomassini:** Resources, Writing – review & editing, Funding acquisition. **Nils Muhlert:** Validation, Supervision, Writing – review & editing. **Ilona Lipp:** Conceptualization, Methodology, Software, Validation, Formal analysis, Data curation, Writing – review & editing, Visualization, Supervision, Project administration.

## Declaration of Competing Interest

The authors declare that they have no known competing financial interests or personal relationships that could have appeared to influence the work reported in this paper.
