# Supplementary material for: A tractometry principal component analysis of white matter tract network structure and relationships with cognitive function in relapsing-remitting multiple sclerosis
Source: Neuroimage Clin. 2022 Mar 24;34:102995. doi: 10.1016/j.nicl.2022.102995 (PMC8958271; doi:10.1016/j.nicl.2022.102995)
Supplement: Supplementary data 2 [file mmc2.docx]

# Appendix 2

**Supplementary analysis 1: Influence of lesions**

To understand whether the presence of lesions has an influence on the component structure that can be seen across white matter tracts, we repeated the analysis presented in the main manuscript but without masking out lesions from the tracts before average diffusivity metrics were extracted. All steps were exactly the same except for the regressing out of predictors of the first tract component. Because this was lesion volume, and the aim of the current analysis was to understand the contribution of lesions to the component structure, this step was omitted. The results presented below are only for MS patients as healthy controls did not have lesions.

A PCA of the four microstructural metrics was conducted based on a significant Bartlett’s test of sphericity (χ^2^(6) = 151.89, *p* <0.001). Based on scree plot inspection and eigenvalues >1, only the first principal component, which explained 60% of variance, was extracted. The component loadings were 0.91 for FA, -0.87 for RD, 0.88 for MWF and 0.20 for MTR, indicating that similarly to the main analyses, FA, RD and MWF were the main contributors to the first metric component.

Component scores of this principal component were calculated for each patient and each tract and correlated. A Bartlett’s test of sphericity showed that a PCA was suitable for tract metrics (χ^2^(780) = 6150.97, *p* <0.001). The scree plot showed one strong principal component that explained 67% of variance, but all of the first four components had an eigenvalue >1 (6%, 4% and 3% variance explained, respectively), and were subjected to a Varimax rotation. After rotation all tracts still loaded positively on rotated tract component (TC) 1. See Figure 1A for the metric and tract correlation matrices and scree plots and Table 2 for the rotated component tract loadings.

To check what anatomical and demographic factors contributed to variance of TC1, simple correlation coefficients were calculated and a linear regression analysis was performed. Lesion volume (r=-0.47), normalised brain volume (r=0.26) and normalised grey matter volume (r=0.26) correlated most highly with TC1 weightings (see Figure 1B). Before the regression analysis was performed, all demographic and anatomical were inputted into a correlation matrix to assess the degree of multicollinearity. Normalised brain volume, normalised grey matter volume and normalised white matter volume corrected highly, so of these variables, only normalised brain volume was included in the regression model. Lesion volume (ß = -0.48, p < 0.001) was found to be the only significant predictor of TC1 (R^2^ = 0.25, *F*(6, 95) = 6.58, *p* < 0.001). Full statistics for this model are presented in Table 1.

The cognitive domains described in the main manuscript were used to understand how tract components based on diffusion metrics from tracts with lesions predicts function across the four cognitive domains. Similarly, to the main analysis, tract components

In MS, tract components did not predict cognitive component (CC) scores to a high degree, nor did demographic and MRI variables (see Table 1). The first cognitive component, CC1, was best predicted by TC1 (ß = 0.43, p = 0.005), TC3 (ß = 0.28, p = 0.021), sex (ß = 0.33, p = 0.014), and NBV (ß = -0.29, p = 0.037), in a model explaining 19% of variance (R^2^ = 0.19, F(10, 91) = 3.37, p < 0.001). The final cognitive component, CC4, was best predicted by age (ß = -0.29, p = 0.011), in a model explaining 18% of the variance (R^2^ = 0.18, F(10, 91) = 3.24, p = 0.001). The regression models for CC2 and CC3 were not significant. Please see Table 1 for full statistical results.

The results of this additional analysis suggest that the presence of lesions in WM tracts adds relatively little additional variance to average diffusion metrics, and this additional variance does not influence the component structure of the white matter in the MS brain.

**Table A.1. Predictors of WM tract covariance and cognitive domains**

| **Model** | **Predictors** | **Model statistics** |
| --- | --- | --- |
| **Unrotated tract component 1** | Age: ß = -0.15, p = 0.16  Sex: ß = 0.01, p = 0.90  Education: ß = -0.04, p = 0.65  ICV: ß = 0.23, p = 0.06  *Lesion volume: ß = -0.48, p < 0.001*  NBV: ß = -0.02, p = 0.88 | R^2^ = 0.25, *F*(6, 95) = 6.58, *p* < 0.001 |
| **CC1: Verbal cognition** | *TC1: ß = 0.43, p = 0.005*  TC2: ß = 0.005, p = 0.972  *TC3: ß = 0.28, p = 0.021*  TC4: ß = -0.05, p = 0.622  Age: ß = -0.002, p = 0.987  *Sex: ß = 0.33, p = 0.014*  Education: ß = 0.7, p = 0.478  ICV: ß = 0.16, p = 0.252  Lesion volume: ß = 013, p = 437  *NBV: ß = -0.29, p = 0.037* | R^2^ = 0.19, F(10, 91) = 3.37, p < 0.001 |
| **CC2: Visuospatial cognition** | TC1: ß = -0.13, p = 0.423  TC2: ß = -0.18, p = 0.193  TC3: ß = -0.06, p = 0.671  TC4: ß = 0.06, p = 0.566  *Age: ß = -0.32, p = 0.009*  Sex: ß = 0.08, p = 0.561  Education: ß = 0.10, p = 0.312  ICV: ß = -0.005, p = 0.974  Lesion volume: ß = -0.21, p = 0.256  NBV: ß = -0.02, p = 0.908 | R^2^ = 0.03, F(10, 91) = 1.35, p = 0.214 |
| **CC3: Information processing** | TC1: ß = 0.15, p = 0.369  TC2: ß = 0.23, p = 0.109  TC3: ß = 0.10, p = 0.449  TC4: ß = 0.10, p = 0.366  Age: ß = 0.20, p = 0.102  Sex: ß = -0.19, p = 0.202  Education: ß = -0.005, p = 0.962  ICV: ß = -0.11, p = 0.450  Lesion volume: ß = 0.27, p = 0.156  NBV: ß = 0.20, p = 0.187 | R^2^ = -0.008, *F*(10, 91) = 0.09, *p* = 0.522 |
| **CC4: Executive function** | TC1: ß = 0.14, p = 0.360  TC2: ß = 0.12, p = 0.338  TC3: ß = 0.03, p = 0.773  TC4: ß = 0.15, p = 0.111  *Age: ß = -0.29, p = 0.011*  Sex: ß = -0.04, p = 0.747  Education: ß = 0.07, p = 0.430  ICV: ß = -0.06, p = 0.677  Lesion volume: ß = -0.08, p = 0.636  NBV: ß = 0.11, p = 0.425 | R^2^ = 0.18, F(10, 91) = 3.24, p = 0.001 |

Significant predictors are presented in italics. Significance threshold *p* < 0.05 applied unless otherwise indicated. Abbreviations: CC = cognitive component, ICV = intracranial volume, NBV = normalised brain volume, NWMV = normalised white matter volume, TC = tract component, WM = white matter

**Table A.2. Tract loadings on each component derived from the tract PCA, after applying Varimax rotation**

| **TC1** | | **TC2** | | **TC3** | | **TC4** | |
| --- | --- | --- | --- | --- | --- | --- | --- |
| **Tract** | **Loading** | **Tract** | **Loading** | **Tract** | **Loading** | **Tract** | **Loading** |
| fma | 0.76 | str_l | 0.89 | cbp_r | 0.79 | mcp | 0.84 |
| or_l | 0.72 | cst_l | 0.84 | cbd_r | 0.74 | ac | 0.30 |
| vof_r | 0.72 | str_r | 0.80 | ac | 0.71 | or_r | 0.30 |
| vof_l | 0.69 | cst_r | 0.77 | cbt_r | 0.70 | ar_r | 0.26 |
| or_r | 0.68 | fa_l | 0.74 | cbt_l | 0.70 | fma | 0.23 |
| mdlf_l | 0.67 | fa_r | 0.72 | cbp_l | 0.69 | mdlf_r | 0.21 |
| ar_l | 0.66 | af_l | 0.71 | uf_l | 0.67 | atr_r | 0.21 |
| ifo_r | 0.65 | slf3_l | 0.71 | fmi | 0.66 | cst_r | 0.21 |
| mdlf_r | 0.65 | af_r | 0.71 | cbd_l | 0.66 | ifo_r | 0.20 |
| ifo_l | 0.64 | slf2_r | 0.70 | uf_r | 0.61 | vof_r | 0.19 |
| ilf_r | 0.62 | slf1_l | 0.69 | atr_l | 0.59 | atr_l | 0.16 |
| ilf_l | 0.57 | slf2_l | 0.67 | atr_r | 0.56 | af_r | 0.14 |
| ar_r | 0.56 | slf3_r | 0.67 | ilf_l | 0.55 | slf3_r | 0.13 |
| slf1_r | 0.55 | atr_l | 0.63 | ifo_l | 0.50 | cbt_r | 0.13 |
| af_r | 0.54 | mdlf_r | 0.59 | ilf_r | 0.47 | ilf_r | 0.12 |
| slf3_r | 0.53 | slf1_r | 0.55 | mdlf_l | 0.46 | fmi | 0.12 |
| slf2_r | 0.52 | ifo_r | 0.53 | fa_l | 0.44 | or_l | 0.12 |
| slf2_l | 0.50 | atr_r | 0.53 | slf1_l | 0.43 | ilf_l | 0.10 |
| cbt_l | 0.49 | or_r | 0.52 | ifo_r | 0.41 | uf_r | 0.08 |
| cbd_l | 0.48 | mdlf_l | 0.51 | slf2_l | 0.40 | slf2_r | 0.08 |
| uf_r | 0.47 | ifo_l | 0.50 | ar_l | 0.40 | str_r | 0.05 |
| af_l | 0.46 | cbd_l | 0.48 | or_l | 0.38 | str_l | 0.05 |
| fa_r | 0.46 | ilf_r | 0.48 | af_l | 0.38 | cbd_r | 0.05 |
| fmi | 0.45 | fmi | 0.46 | fma | 0.37 | slf1_r | 0.04 |
| cbt_r | 0.42 | or_l | 0.46 | slf3_l | 0.36 | cbt_l | 0.04 |
| slf3_l | 0.41 | uf_r | 0.44 | fa_r | 0.35 | ifo_l | 0.03 |
| cbd_r | 0.39 | ar_r | 0.43 | vof_l | 0.34 | mdlf_l | 0.03 |
| slf1_l | 0.39 | uf_l | 0.42 | slf1_r | 0.33 | cbd_l | 0.02 |
| uf_l | 0.36 | cbd_r | 0.40 | mdlf_r | 0.32 | slf3_l | -0.01 |
| atr_r | 0.35 | cbp_l | 0.36 | af_r | 0.31 | cst_l | -0.01 |
| cbp_l | 0.35 | fma | 0.29 | slf3_r | 0.29 | cbp_r | -0.02 |
| fa_l | 0.33 | cbp_r | 0.29 | slf2_r | 0.26 | fa_r | -0.03 |
| atr_l | 0.29 | ilf_l | 0.25 | or_r | 0.26 | vof_l | -0.03 |
| cst_r | 0.28 | ar_l | 0.22 | vof_r | 0.23 | af_l | -0.03 |
| cbp_r | 0.25 | vof_l | 0.19 | ar_r | 0.22 | cbp_l | -0.03 |
| str_r | 0.25 | cbt_l | 0.19 | cst_r | 0.18 | slf2_l | -0.04 |
| mcp | 0.19 | vof_r | 0.18 | str_r | 0.18 | slf1_l | -0.04 |
| str_l | 0.14 | cbt_r | 0.16 | cst_l | 0.17 | uf_l | -0.05 |
| ac | 0.06 | ac | 0.06 | str_l | 0.15 | fa_l | -0.08 |
| cst_l | 0.03 | mcp | 0.04 | mcp | 0.13 | ar_l | -0.16 |

Abbreviations: ac = anterior commissure; af = arcuate fasciculus; ar = acoustic radiation; atr = anterior thalamic radiation; cbd = cingulum subsection, dorsal; cbp = cingulum subsection, peri-genual; cbt = cingulum subsection, temporal; cst = corticospinal tracr; fa = frontal aslant; fma = forceps major; fmi = forceps minor; ifo = inferior fronto-occipital fasciculus; ilf = inferior longitudinal fasciculus; mcp = middle cerebellar peduncle; mdlf = middle longitudinal fasciculus; or = optic radiation; slf1-3 = superior longitudinal fasciculus 1-3; str = superior thalamic radiation; uf = uncinate fasciculus; vof = vertical occipital fasciulus. Left and right hemisphere tracts are denoted with _l and _r, respectively.


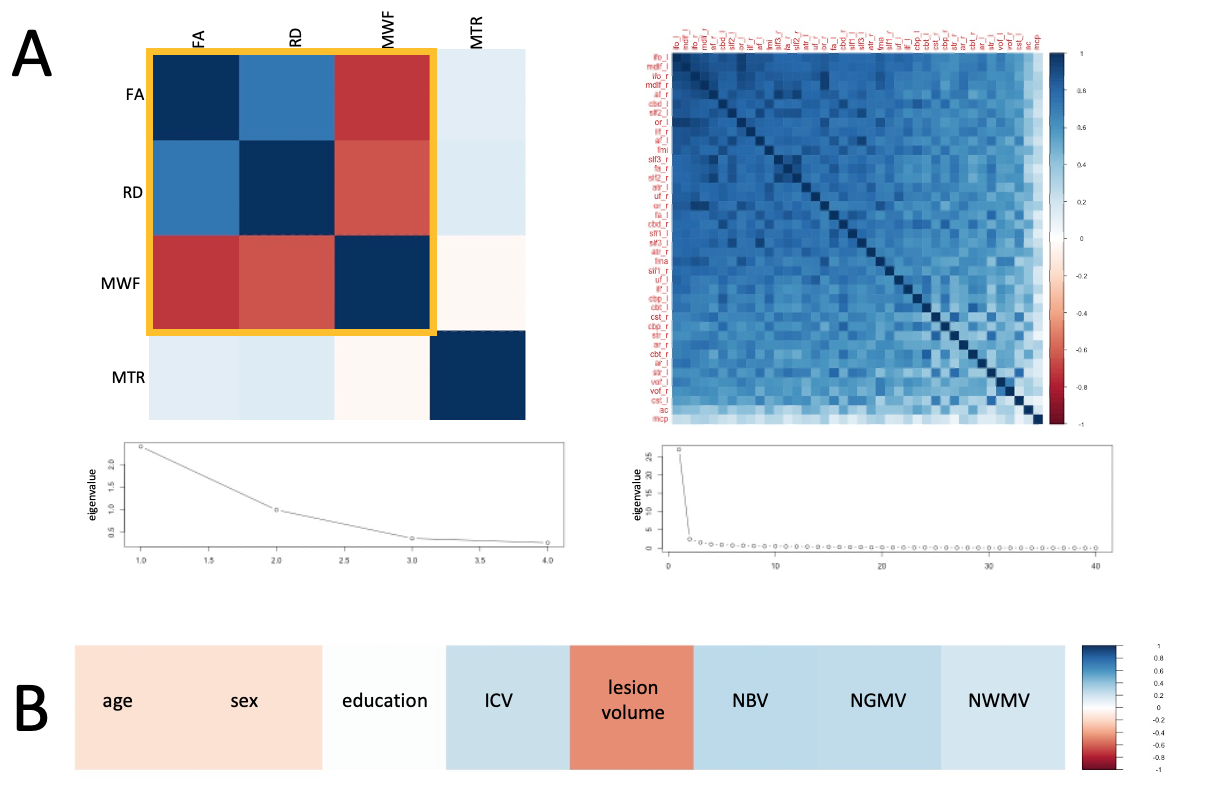


**Figure A.1. Metric and tract principal component analysis in people with multiple sclerosis**

Figure 1A shows the correlation matrices and scree plots for the PCA ran on the four white matter microstructural metrics (left) and the white matter tracts based on the first component from the metric PCA (right). Those metrics marked with a yellow line load most on principal component 1. All tracts loaded positively on tract principal component 1. Figure 1B shows correlations between rotated tract principal component 1 (TC1) and demographic and anatomical variables. Abbreviations: FA = fractional anisotropy; RD = radial diffusivity; MWF = myelin water fraction; MTR = magnetisation transfer ratio; ICV = intracranial volume; NBV = normalised brain volume; NGMV = normalised grey matter volume; NWMV = normalised white matter volume
