# Supplementary material for: A tractometry principal component analysis of white matter tract network structure and relationships with cognitive function in relapsing-remitting multiple sclerosis
Source: Neuroimage Clin. 2022 Mar 24;34:102995. doi: 10.1016/j.nicl.2022.102995 (PMC8958271; doi:10.1016/j.nicl.2022.102995)
Supplement: Supplementary data 3 [file mmc3.docx]

# Appendix 3

**Supplementary analysis 2: Effect of diffusion measures, including mean FA from TBSS, on regression models of cognitive components**

To understand how informative tract components are on cognitive components relative to demographic and anatomical variables and more conventional diffusion measures, the regression models were repeated adding mean FA from each participant’s TBSS skeleton. TBSS was performed in FSL (Smith *et al.*, 2006), and the distribution of FA within each participant’s skeletonised FA map was visually assessed for normality through histogram inspection. Following confirmation of normality, a mean FA value from the whole skeleton was extracted for each participant and added to the regression model for assessing predictive variables of variance in cognitive component scores.

First, we correlated FA from TBSS to our main tract component, TC1, to understand whether, and to what extent they are related. This correlation was significant, but of weak to moderate strength (r=0.32, p = 0.001). Thus, they both reflect an aspect of whole brain microstructural integrity, but also differ in ways that are important to understand. Particularly, their respective importance to cognitive function in multiple sclerosis is of key interest, to understand if one is more sensitive to cognitive impairment than the other. By assessing their correlations with cognitive domains, and whether they are significant predictors of cognitive domain variance, we can better understand if the tract component obtained adds more explanatory power to cognitive function than more conventional diffusion metrics.

For all multiple regression models presented below, the adjusted R-squared is reported, to adjust for the number of predictors in the model.

Cognitive component (CC) 1 was best predicted by tract component (TC) 1 (ß = 0.26, p = 0.044), sex (ß = 0.36, p = 0.010) and normalised brain volume (ß = -0.34, p = 0.022), which together explained 16% of variance (R^2^ = 0.16, F(11, 90) = 2.81, p = 0.003). CC4 was best predicted by age (ß = -0.27, p = 0.019) in a model explaining 17% of variance (R^2^ = 0.17, *F*(11, 90) = 2.87, *p* = 0.003). The models for CC2 and CC3 were not significant. Please see Table 1 for full statistics. Thus, FA from TBSS was not a significant predictor of any cognitive component, and including it led to a minor decrease in the variance explained in CC1 (16% compared to 17% without it, see main manuscript) and CC4 (17% compared to 18% without it, see main manuscript). Thus, the first tract component is more sensitive to variance in verbal cognition than FA from TBSS, but neither appears to have high sensitivity to the other cognitive domains.

Univariate correlation tests were also performed to understand the relationships between cognitive domains and the variables included in regression models better. Some variables which did correlate with cognitive domains were not significant predictors in regression models aimed at explaining cognitive variance. Of note, both normalised brain volume (r=0.37, p < 0.001) and FA from TBSS (r=0.38, p < 0.001) correlated significantly with cognitive domain 4, executive function, but were not significant predictors in the regression model (ß = 0.13, p = 0.390 and ß = 0.05, p = 0.809, respectively). Please see table 1 for full statistics.

It should be noted that due the exploratory nature of these supplementary analyses, no multiple comparison correction was applied. Therefore, given the many statistical analyses performed, the results need to be interpreted with caution and awareness of the possibility of the presence of type 1 errors.

**Table A.1. Predictors and correlates of cognitive domains**

|  | **CC1: verbal cognition** | | | **CC2: visuospatial cognition** | | | **CC3: Information processing** | | | **CC4: Executive function** | | |
| --- | --- | --- | --- | --- | --- | --- | --- | --- | --- | --- | --- | --- |
| **Variable** | **Multiple regression** | | **Univariate correlation** | **Multiple regression** | | **Univariate correlation** | **Multiple regression** | | **Univariate correlation** | **Multiple regression** | | **Univariate correlation** |
|  | **Beta co-efficients** | **Model statistics** |  | **Beta co-efficients** | **Model statistics** |  | **Beta co-efficients** | **Model statistics** |  | **Beta co-efficients** | **Model statistics** |  |
| **TC1** | *ß = 0.26, p = 0.044* | *R^2^ = 0.16, F(11, 90) = 2.81, p = 0.003* | r=-0.73, p = 0.22 | ß = 0.001, p = 0.995 | R^2^ = 0.04, *F*(11, 90) = 1.35, *p* = 0.210 | r=0.05, p = 0.62 | ß = -0.02, p = 0.866 | R^2^ = 0.07, *F*(11, 90) = 1.68, *p* = 0.090 | r=0.10, p = 0.32 | -0.03, p = 0.801 | *R^2^ = 0.17, F(11, 90) = 2.87, p = 0.003* | r=0.09, p = 0.36 |
| **TC2** | ß = -0.13, p = 0.278 |  | r=-0.11, p = 0.26 | ß = -0.12, p = 0.343 |  | r=-.10, p = 0.32 | ß = 0.02, p = 0.883 |  | r=0.11, p = 0.28 | 0.03, p = 0.786 |  | r=0.10, p = 0.31 |
| **TC3** | ß = 0.10, p = 0.326 |  | r=0.13, p = 0.17 | ß = 0.09, p = 0.452 |  | r=0.08, p = 0.44 | ß = 0.17, p = 0.140 |  | *r=0.23, p = 0.02* | 0.14, p = 0.187 |  | r=0.16, p = 0.12 |
| **TC4** | ß = 0.07, p = 0.496 |  | r=0.14, p = 0.15 | ß = -0.10, p = 0.327 |  | r=-0.07, p = 0.46 | ß = -0.20, p = 0.055 |  | r=-0.19, p = 0.05 | 0.02, p = 0.799 |  | r= 0.04, p = 0.71 |
| **Age** | ß = 0.005, p = 0.966 |  | r=0.09, p = 0.36 | *ß = -0.32, p = 0.009* |  | *r=-0.30, p = 0.003* | ß = 0.20, p = 0.092 |  | r=0.07, p = 0.48 | *-0.27, p = 0.019* |  | *r=-0.37, p < 0.001* |
| **Sex** | *ß = 0.36, p = 0.010* |  | *r=0.34, p < 0.001* | ß = 0.10, p = 0.487 |  | r=0.07, p = 0.49 | ß = -0.14, p = 0.329 |  | r=-0.11, p = 0.27 | -0.02, p = 0.856 |  | r=-0.02, p = 0.82 |
| **Education** | ß = 0.05, p = 0.633 |  | r=0.09, p = 0.39 | ß = 0.10, p = 0.353 |  | r=0.17, p = 0.09 | ß = -0.04, p = 0.705 |  | r=-0.04, p = 0.66 | ß = 0.07, p = 0.468 |  | r=0.14, p = 0.15 |
| **ICV** | ß = 0.10, p = 0.447 |  | r=-0.16, p = 0.11 | ß = 0.01, p = 0.941 |  | r=-0.06, p = 0.57 | ß = -0.13, p = 0.366 |  | r=0.07, p = 0.50 | -0.03, p = 0.844 |  | r=0.04, p = 0.73 |
| **Lesion volume** | ß = -0.11, p = 0.559 |  | r=-0.08, p =0.42 | ß = -0.07, p = 0.717 |  | r=-0.04, p = 0.68 | ß = 0.25, p = 0.199 |  | r=-0.04, p = 0.66 | ß = -0.20, p = 0.270 |  | *r=-0.30, p = 0.002* |
| **NBV** | *ß = -0.34, p = 0.022* |  | r=-0.07, p =0.47 | ß = -0.03, p = 0.831 |  | r=0.12, p = 0.22 | ß = 0.16, p = 0.316 |  | r=0.13, p = 0.19 | ß = 0.13, p = 0.390 |  | *r=0.37, p < 0.001* |
| **TBSS FA** | ß = 0.18, p = 0.426 |  | r=0.12, p = 0.24 | ß = -0.04, p = 0.864 |  | r=0.05, p = 0.64 | ß = 0.32, p = 0.172 |  | r=0.19, p = 0.05 | ß = 0.05, p = 0.809 |  | *r=0.38, p < 0.001* |

Results reaching statistical significance are presented in italics. Significance threshold *p* < 0.05 applied unless otherwise indicated. Abbreviations: CC = cognitive component, FA = fractional anisotropy, ICV = intracranial volume, NBV = normalised brain volume, NWMV = normalised white matter volume, TBSS = tract based spatial statistics, TC = tract component, WM = white matter
